# Micronuclei detection in oral cytologic smear: does it add diagnostic value?

**DOI:** 10.1186/s43046-023-00188-x

**Published:** 2023-09-25

**Authors:** Alaa Elnaggar, Gihane Madkour, Neveen Tahoun, Ayman Amin, Fat’heya M. Zahran

**Affiliations:** 1 Oral Medicine and Periodontology Department, Faculty of Dentistry, Cairo University, Cairo, Egypt; 2 National Cancer Institute, Cairo University, Cairo, Egypt

**Keywords:** Micronuclei, Exfoliative cytology, Oral potentially malignant lesions, Oral cancer

## Abstract

**Background:**

Screening and early diagnosis of oral squamous cell carcinoma (OSCC) are directly associated with increased survival rate and improved prognosis. Noninvasive diagnostic tools have been implemented in the early detection as toluidine blue staining, optical imaging, and oral cytology. This study aimed to assess and compare the presence of micronuclei (MN) in oral exfoliative cytology of healthy controls, subjects exposed to high-risk factors for oral cancer, subjects with oral potentially malignant lesions (OPMLs), and those with malignant oral lesions.

**Subjects and methods:**

A total number of 92 subjects were divided into 46 healthy controls with no oral mucosal lesions (23 with no evidence of cancer risk factors and 23 with cancer risk factors), 23 with OPMLs and 23 with oral malignant lesions. All the 92 participants were subjected to cytological sampling for detection of MN. The final diagnosis of the oral lesions was confirmed by the histopathological picture and compared to the cytological results.

**Results:**

The results showed that the diagnostic accuracy of MN was higher in OPMLs group (95.2%). The sensitivity of MN test in malignant group was much lower (52.2%); however, all the cytological criteria of malignancy were markedly detected as compared to the OPMLs group.

**Conclusions:**

Conventional oral cytology supported by MN is highly beneficial as adjunctive tool in the screening for early detection of dysplastic oral lesions.

**Supplementary Information:**

The online version contains supplementary material available at 10.1186/s43046-023-00188-x.

## Introduction

Oral cancer is the sixth most prevalent cancer worldwide, and about 90% of oral cancer is OSCC. OSCC mostly presents with a highly aggressive course, tendency for lymph node metastasis, and poor prognosis [1]. Based on the world cancer data at 2000, the survival rate is greatly low, about 40%, and it can rise above 80% when it is detected at the early stages (I and II) [2]. A wide range of health-related behaviors are associated with oral cancer such as smoking, excessive alcoholic drinking, areca nut chewing, HPV infection, and poor oral health [3]. The World Health Organization (WHO) assigned the oral mucosal disorders that have high risk of malignant transformation as OPMLs. Although most of OPMLs are displayed as asymptomatic conditions, the rate of oncogenic abnormalities differs in accordance to patients’ risk factors and lesions’ characteristics as size, consistency, and site [4]. The first step in oral cancer screening is performed through visual and tactile clinical examination. Actually, the main drawback of clinical visualization is subjectivity, so adjunctive tools have been introduced to enhance the visibility of neoplastic lesions as the use of toluidine blue vital staining and autofluorescence imaging. The cytological examination adds benefits to the diagnostic pathway as it has the capability to distinguish between benign and malignant cells in reference to the morphological and the cellular features. Furthermore, it is a simple, rapid, noninvasive technique that is well accepted by the patient and could be regarded as a promising modality for oral cancer mass screening purposes [5]. However, it is not a substitute for scalpel biopsy; rather, it just captures the nature of the lesion [6]. On the other hand, it has been reported that MN formation is an early cytological consequence of chromosomal aberrations induced by genotoxic agents. MN are produced within epithelial cells either from unrepaired DNA breaks or defects in the spindle apparatus and chromosomal segregation machinery [7]. They are mainly developed at the basal cell layer of epithelium. The cells carrying the MN are shed as exfoliated cells on maturation and appear at the superficial layer with higher frequency [8]. MN are regarded by some previous authors as a useful biomarker for screening genotoxic damage and detection of chromosomal aberrations in both OPMLs and malignant lesions [9]. It has even been postulated that the diagnostic quality of oral cytology is enhanced utilizing MN detection in the cytological assessment of OPMLs especially in clinically benign-looking lesions [10]. Hence, the aim of the study was to evaluate the diagnostic value of MN detection in exfoliative cytology for the assessment of OPMLs and malignant lesions. Also, the cytological criteria of dysplasia and malignancy and its correlation with MN were assessed as a secondary outcome.

## Subjects and methods

This is phase II case-comparant diagnostic accuracy study. Its protocol is registered on ClinicalTrials.gov under identifier NCT04955197. The study was approved by the Research Ethics Committee (CREC), Faculty of Dentistry-Cairo University, with an approval number 19–9-22. The purpose of the study was described in details for the subjects before participation, followed by obtaining an informed consent.

The study was held at the Faculty of Dentistry, Cairo University and the National Cancer Institute from December 2020 to November 2021. The included study participants were enrolled in the study in a consecutive order of all patients evaluated for eligibility at the study location and satisfying the inclusion criteria. A total sample of 92 adult patients were divided into four groups: 23 participants with clinically normal oral mucosa and no evidence of any cancer risk factor (family history of cancer, cigarettes smoking and shisha, alcohol consumption), 23 participants with clinically normal oral mucosa and evidence of cancer risk factors, 23 patients with OPMLs, and 23 patients recently diagnosed with OSCC and not receiving yet any treatment. The exclusion criteria in the control groups were as follows: systemic disease and recent viral infection.

### Questionnaire administration

Face-to-face interviews were performed with the study participants to obtain detailed information regarding their demographics, medical status, and cancer risk factors.

### Clinical examination

All patients were subjected to a detailed intraoral and extraoral examination according to the National Institute of Dental and Craniofacial Research (2013) [11]. Subjects with oral lesions were assessed as follows:

- ◦ OSCC group: The tumor site was recorded, and grade was obtained after histopathologic examination.
- ◦ OPML group: The site and size of the lesion were recorded. The type of oral lichen planus was mentioned as; atrophic, erosive or papular. While, the leukoplakia cases were described as homogenous, non-homogenous, or proliferative verrucous leukoplakia.
- ◦ For control groups, no oral lesions were detected on conventional tactile and visual examination.

### Index test

#### Oral exfoliative cytology procedure

All the cytological samples were taken at the same day of tissue biopsy in both OPMLs and malignant groups to avoid disease progression bias. The mechanical scraping of the oral mucosa was done by the use of cyto-brush. The sample was obtained from the mucosal lesion of the compared groups. While the buccal mucosa was the target of the cytological sampling in the risk factors and the control groups as it is an easily accessible tissue for sampling in a minimally invasive manner and does not cause any stress on study subjects, additionally, most of the encountered lesions were located at the buccal mucosa. For lesions that affect multiple sites — seen in the OPML group — the most suspicious and representative site was selected.

The sites of cytological smear were cleaned with a sterile cotton before sampling to remove viscous saliva covering the lesion and any debris from the oral cavity. Area was anesthetized with topical lignocaine spray.

The head of the cyto-brush was twisted to be perpendicular to the handle to facilitate the scrapping action and to direct pressure application. The brush was firmly applied with force against the mucosal lesion, and then pressure was applied while rolling the brush over the lesion area for 10 full turns until the bristles curled or pin point bleeding spot was evident [12]. The collected cells were smeared onto a labelled glass slide by rolling the cyto-brush in a continued motion from one end of the slide to the other (technique is shown in supplementary file 1). The slide was immediately immersed in 95% ethanol for fixation and stained with Papanicolaou stain (Pap stain) [13]. The Pap stain is the widely used cytological staining technique and is the preferred modality for demonstrating MN. It provides a distinct nuclear and cellular staining with highly defined nuclear details and cytoplasmic transparency [14].

### Interpretation

The samples were evaluated and interpreted in reference to the alterations at the cytological level defined by the cytological features of malignancy and MN. The slides were completely screened by the cytopathologist for identification of any cytological abnormalities (negative or positive).

### The cytological criteria of dysplasia or malignancy

The general features of malignancy in the cytological slides were reported as follows: high cellularity, increased nuclear/cytoplasmic ratio, nuclear hyperchromasia, discohesiveness of cells, nuclear membrane abnormalities, cellular pleomorphism, anisonucleosis, prominent nucleoli, and irregular mitoses.

**Fig. 1:**
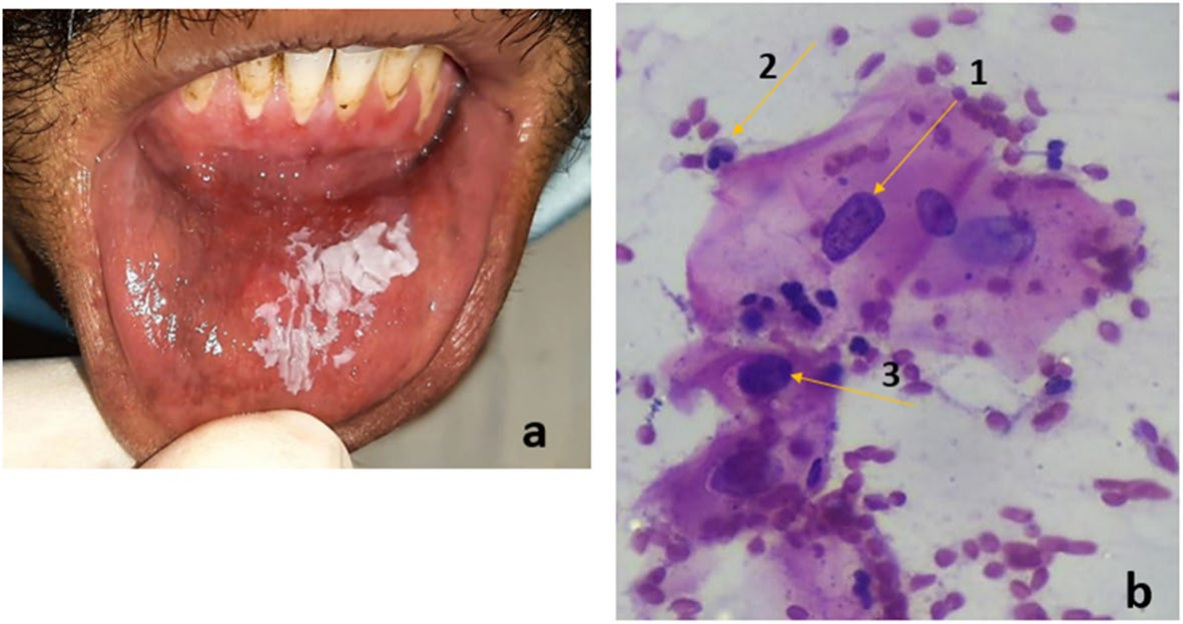
A 34-year-old male patient, medically free, smoker (packet/day/10 years), reported snuff-dipping habit presented with **a** leukoplakia histopathology revealed mild dysplasia, **b** cytological picture with cellular pleomorphism, arrow (1) MN, arrow (2) anisonucleosis, arrow (3) hyperchromasia

**Fig. 2:**
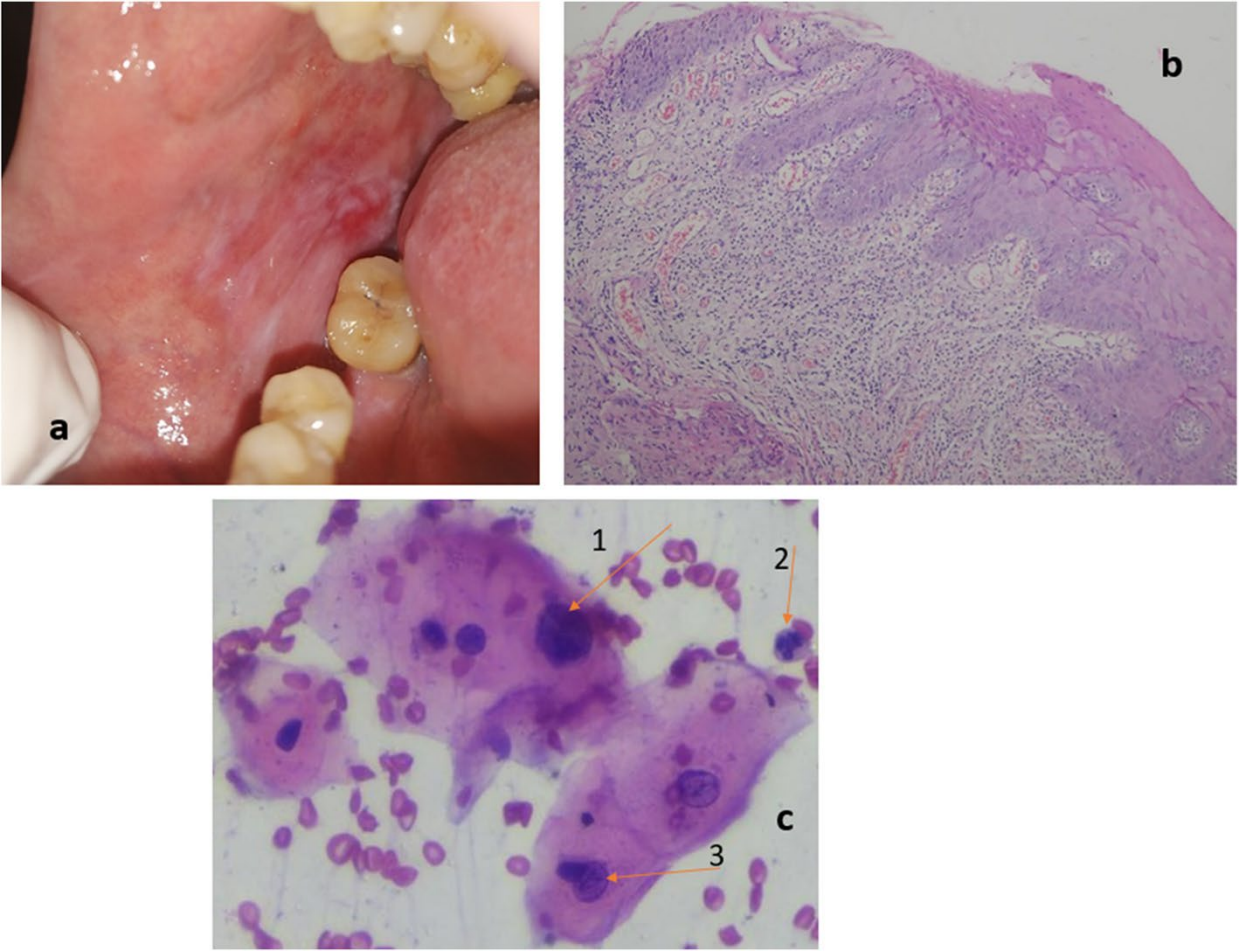
A 48-year-old female diabetic patient presented with (**a**) erosive OLP, (**b**) histopathology moderate dysplasia, (**c**) cytological features arrow (1) increased N/C ratio, hyperchromasia, arrow (2) anisonucleosis, arrow (3) MN

**Fig. 3:**
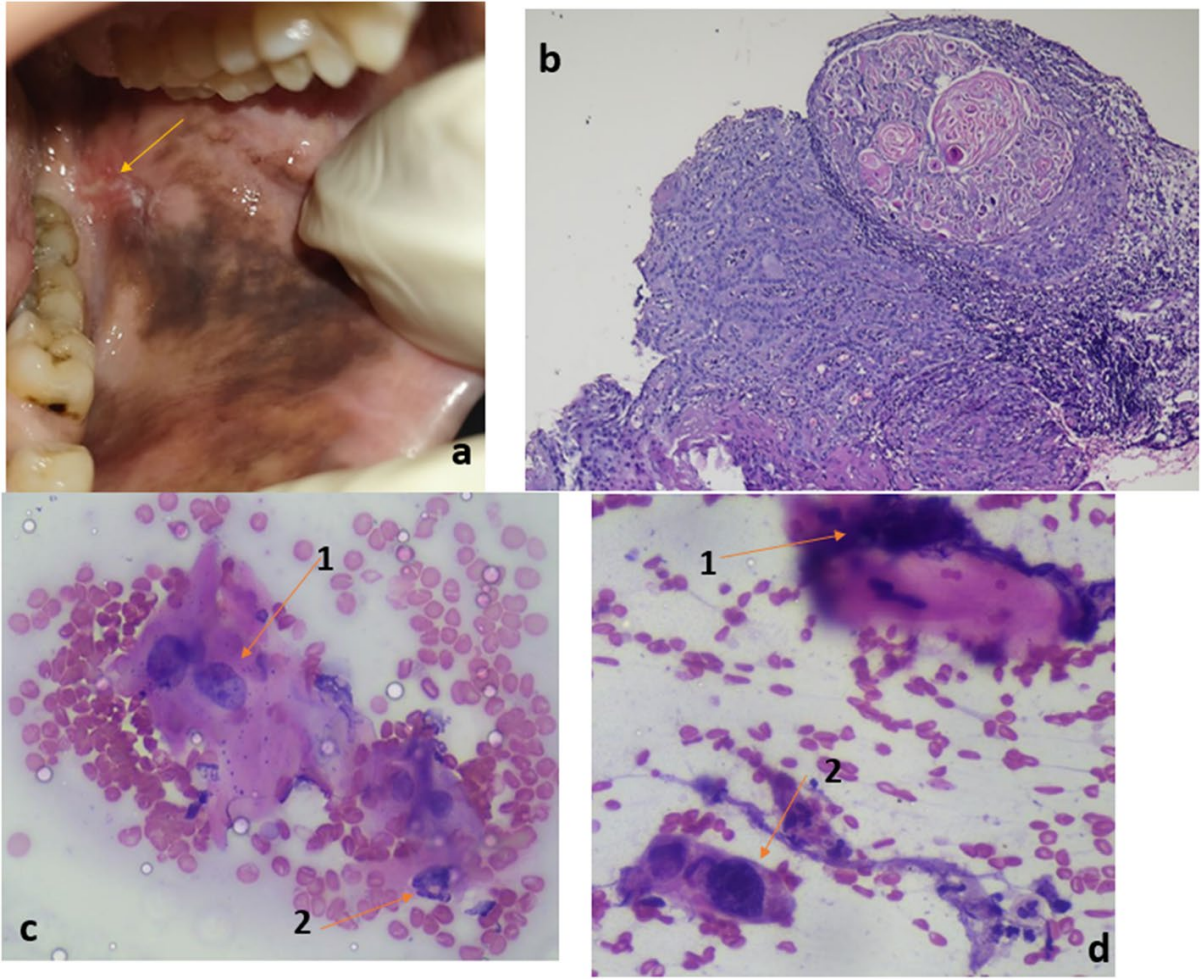
A 33-year-old female patient medically free, smoker (pack/day/2 years), presented with (**a**) atrophic OLP biopsy revealed (**b**) microinvasive SCC, (**c**) cytological picture showing cellular pleomorphism, arrow (1) MN, arrow (2) anisonucleosis, figure (**d**) arrow (1) hyperchromasia, arrow (2) increased N/C ratio

**Fig. 4:**
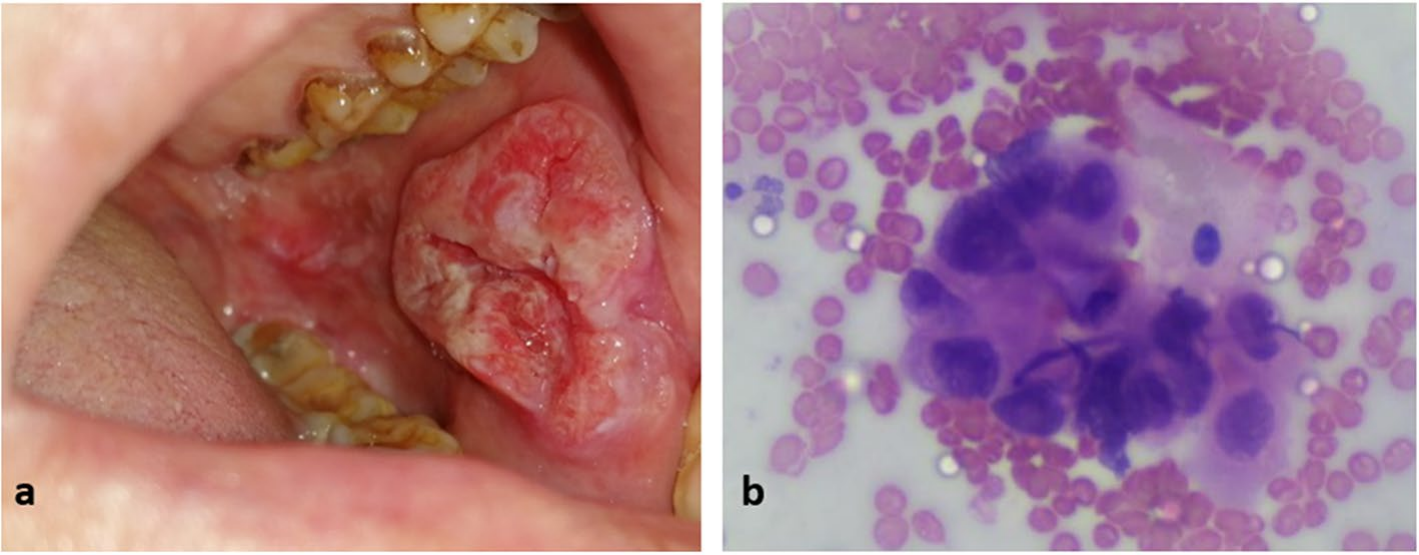
A 66-year-old female patient, medically free, positive family history of cancer, presented with exophytic lesion (**a**) histopathology revealed SCC grade II, T2, and N1, cytological picture (**b**) cellular pleomorphism, increased N/C ratio, irregular nuclear membrane, and hyperchromasia

**Fig. 5:**
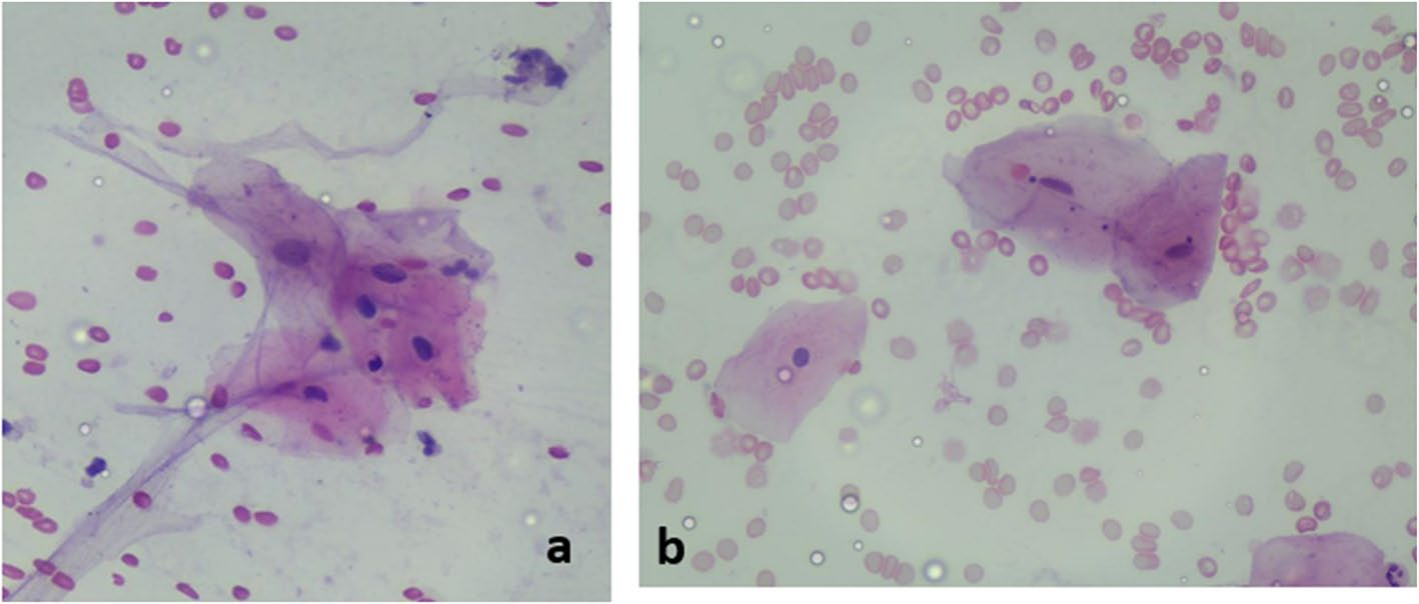
**a** Normal cytology of risk factors group. **b** Normal cytology of control group

Assessment of cytological features was performed on the basis of individual cells along with comparison between different cells. The sample must have a sufficient number of well-preserved cells (at least 30 well-preserved cells) [15]. The uniformity of the features was recorded as benign conditions Fig. 1, whereas pleomorphism was described as dysplasia/malignancy Figs. 2, 3,  4 and 5 [16].

#### Micronuclei

For a sample to be suitable for analysis, it has to meet the following criteria specified by Tolbert et al. (1991) which are as follows [17]:

1. Intact cytoplasm and relatively flat cell position
2. Little or no overlap with adjacent cells
3. Little or no debris
4. Nucleus normal and intact, nuclear perimeter smooth and distinct
5. Fields having at least 1000 epithelial cells

The recommended criteria for the identification of a micronucleus were described by Tolbert et al. (1991) as follows [17]:

1. Rounded smooth perimeter
2. Less than one-third the diameter of the associated nucleus
3. Staining intensity similar to that of the nucleus
4. Texture similar to that of nucleus
5. Same focal plane as nucleus
6. The absence of overlap with, or bridge to, the nucleus

Cells with structures completely fulfilling the abovementioned MN criteria are counted high certainty. Those with objects slightly deficient in MN criteria 3, 4, and 5 and satisfying all of the other criteria are regarded medium certainty. Those assigned medium or high certainty are recorded as MN [17].

The cellular evaluation was performed using optic microscope with magnification (10 × 10) and (40 × 10), and the presence of micronuclei in all subjects was recorded (yes/no). Slides with at least 30 well-preserved cells (i.e., not obscured by blood or exudate or necrosis) are considered adequate for cytological evaluation.

Overlapping or clumped cells were excluded from the analysis. Also, hypocellularity in the slides was removed from analysis. Only cells with intact cytoplasmic border were included in the MN assessment.

The present study recorded MN as frequency (yes/no) rather than count (mean ± SD) in other studies. In reference to the systematic review and meta-analysis by de Geus et al. (2018), wide variations in MN score were observed among different studies. The baseline MN frequency was one of the most significant variable; the calibration of the upper limit of the baseline was not accurately estimated for a given population [18].

### The histopathological assessment

Scalpel biopsy either incisional or excisional was taken from the highly suspicious areas in the OPMLs group. The histopathological picture is still the gold standard utilized for confirmation of the diagnosis and evaluation of the dysplastic changes [14].

OPML included leukoplakia diagnosed clinically and histologically or oral lichen planus diagnosed according to the modified WHO diagnostic criteria [19].

The dysplasia in the OPMLs was graded in reference to the squamous intraepithelial neoplasia/dysplasia (SIN/dysplasia) classification (2005) where mild dysplasia (SIN1) was recorded as low-grade dysplasia, while moderate dysplasia (SIN2), as well as severe dysplasia (SIN3), was categorized as high-grade dysplasia [20]. The OSCC was categorized based on Border’s classification into well differentiated (grade I), moderately differentiated (grade II), and poorly differentiated (grade III) [21].

### Statistical methods

The sample size was calculated based on the previous work by Upadhyay et al. (2019) [22]. The statistical test used was the independent *t*-test with the power of the study 0.80 and level of significance of 0.05. Numerical data were presented as mean and standard deviation values. Categorical data were presented as frequency and percentage values and were analyzed using Fisher’s exact test followed by multiple pairwise comparisons using *z*-tests with Bonferroni correction. Diagnostic accuracy was assessed using ROC curve analysis. The significance level was set at *p* ≤ 0.05 within all tests. Statistical analysis was performed with R statistical analysis software version 4.1.2 for Windows [23].

## Results

The baseline demographic data of the study groups are shown in Table 1. The cancer risk factors among OPMLs and malignant groups are demonstrated in Table 2.

**Table 1:** Demographic data of the study groups

Parameter		Control	Risk	Premalignant	Malignant
**Sex**					
**Male**	**n**	6	11	10	16
	**%**	26.1%	47.8%	43.5%	69.6%
**Female**	**n**	17	12	13	7
	**%**	73.9%	52.2%	56.5%	30.4%
**Age (years)**	**Mean ± SD**	41.83 ± 15.43	39.35 ± 12.10	46.57 ± 8.89	53.00 ± 10.50

**Table 2:** Cancer risk factors in PMLs and malignant group

Parameter		OPMLs	Malignant	*p*-value
**Family history**				
**No**	**n**	13	17	0.353
	**%**	56.5%	73.9%	
**Yes**	**n**	10	6	
	**%**	43.5%	26.1%	
**Cigarette smoking**				
**No**	**n**	11	13	0.768
	**%**	47.8%	56.5%	
**Yes**	**n**	12	10	
	**%**	52.5%	43.5%	

The most prevalent site in OPMLs was the buccal mucosa by a percentage of 78.3%, while OSCC was found to be more common at the tongue with a percentage of 43.5%. The clinical diagnosis of OPMLs was found to be 52% oral lichen planus (OLP), and the plaque OLP was the most common type (66.6%). Oral leukoplakia was 39%, while oral lichenoid reaction was the lowest OPMLs with a percentage of 4.3%. Dysplasia was reported in 56.5% of OPMLs patients; mild dysplasia was the highest prevalent (77%) Fig. 1 followed by moderate dysplasia (15.3%) Fig. 2 and microinvasive SCC (7.6%) Fig. 3. Regarding the malignant group, moderately differentiated SCC grade II was the highest percent (65.2%) Fig. 4, followed by poorly differentiated SCC grade III (21.7%) and well-differentiated SCC grade I (13%).

**Fig. 6:**
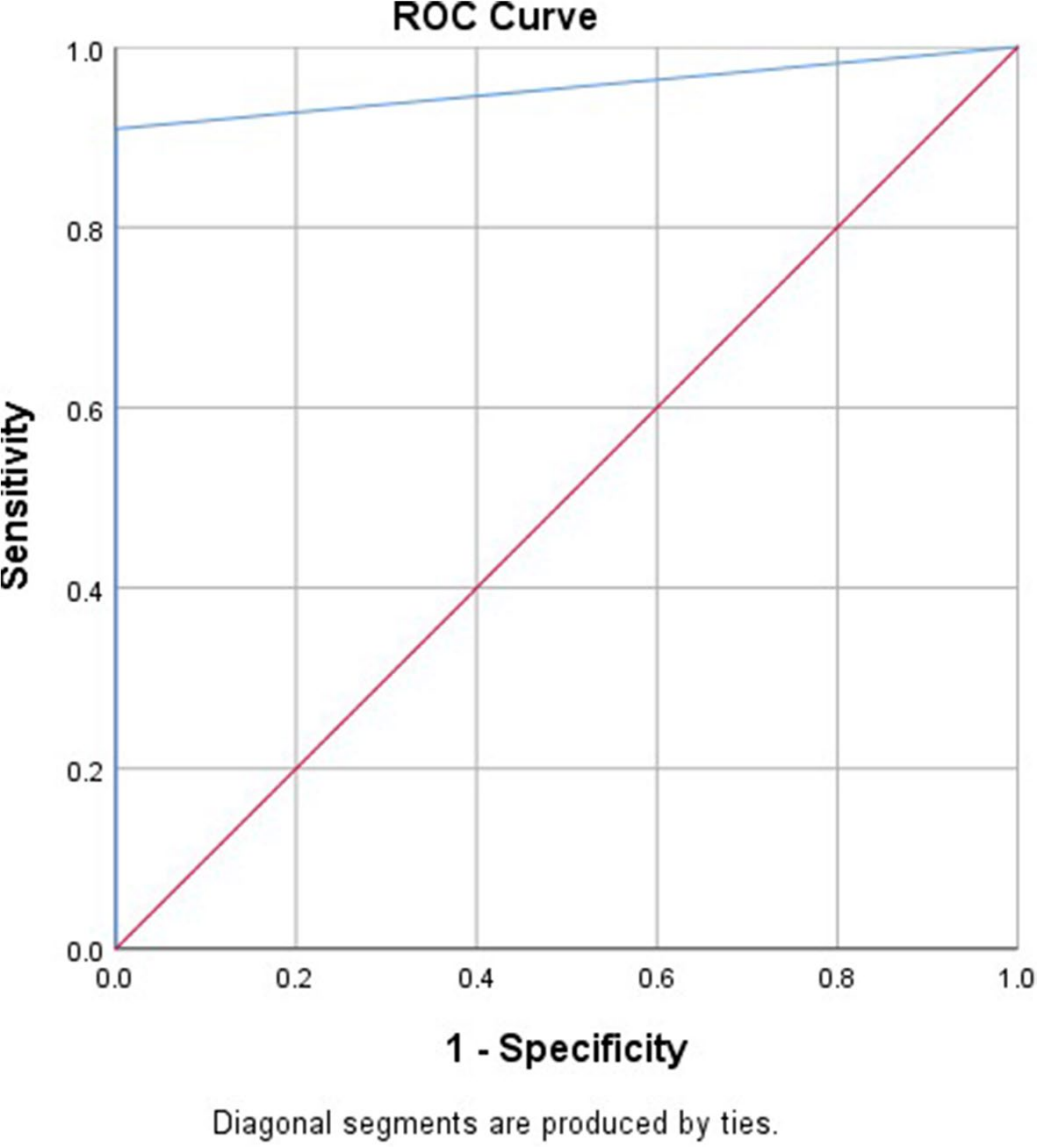
ROC curve MN count in the premalignant group

Cytological sample analysis of both control and risk factors groups showed normal cytological appearance with neither any criteria of malignancy nor MN were detected. In the OPML group, two samples were excluded from the comparative analysis due to the inadequacy of smears (being hypo-cellular). MN were seen in 21 cases in the OPML group and in 23 cases in the OSCC group. The parameters of MN accuracy of both compare groups are presented in Table 3 and Fig. 6.

**Table 3:** Accuracy of MN

Parameter	Premalignant (*n* = 21)	Malignant (*n* = 23)
True positive		
n	10	12
%	100%	100%
True negative		
n	10	0
%	90.9%	0%
False positive		
n	0	0
%	0%	0%
False negative		
n	1	11
%	9.1%	100%
Sensitivity	90.9%	52.2%
95% CI	58.72 to 99.77%	30.59 to 73.18%
Specificity	100%	NA
95% CI	69.15 to 100.00%	
Positive predictive value	100%	100%
95% CI	65–100%	69.8 to 100%
Negative predictive value	90.9%	0%
95% CI	60.68 to 98.48%	0 to 32%
Diagnostic accuracy	95.2%	52.2%
95% CI	76.18 to 99.88%	30.59 to 73.18%
AUC (95% CI)	0.955 (0.866–1)	NA

The cellular and nuclear features of malignancy for both OPMLs and malignant groups were clarified in Table 4.

**Table 4:** Cytological features in OPMLs and malignant groups

Parameter	OPMLs	Malignant	*p*-value
High cellularity			
No	54.5%	4.3%	0.002*
Yes	45.5%	95.7%	
Increased NC ratio			
No	81.8%	65.2%	0.437
Yes	18.2%	34.8%	
Nuclear hyperchromasia			
No	36.4%	4.3%	0.029*
Yes	63.6%	95.7%	
Discohesiveness of cells			
No	36.4%	8.7%	0.070
Yes	63.6%	91.3%	
Membrane abnormalities			
No	72.7%	56.5%	0.465
Yes	27.3%	43.5%	
Prominent nucleoli			
No	9.1%	47.8%	0.053
Yes	90.9%	52.2%	
Cellular pleomorphism			
No	0.0%	17.4%	0.280
Yes	100.0%	82.6%	
Irregular mitosis			
No	90.9%	65.2%	0.214
Yes	9.1%	34.8%	
Anisonucleosis			
No	9.1%	30.4%	0.227
Yes	90.9%	69.6%	

^*^Significant (*p* ≤ 0.05)

For all premalignant cases, there was no significant association between the presence of MN and different cytological features (*p* > 0.05). For the malignant group, there was a significant association between MN and the presence of increased NC ratio (*p* = 0.004), membrane abnormalities (*p* = 0.006), irregular mitosis (*p* = 0.004), and anisonucleosis (*p* = 0.004) (Table 5). For other features, the association was not statistically significant (*p* > 0.05).

**Table 5:** Association between MN count and cytological features in malignant group

Parameter		MN (no)	MN (yes)	*p*-value
**Increased NC ratio**				
**No**	**n**	11	4	0.004*
	**%**	100.0%	33.3%	
**Yes**	**n**	0	8	
	**%**	0.0%	66.7%	
**Membrane abnormalities**				
**No**	**n**	10	3	0.006*
	**%**	90.9%	25.0%	
**Yes**	**n**	1	9	
	**%**	9.1%	75.0%	
**Irregular mitosis**				
**No**	**n**	11	4	0.004*
	**%**	100.0%	33.3%	
**Yes**	**n**	0	8	
	**%**	0.0%	66.7%	
**Anisonucleosis**				
**No**	**n**	7	0	0.004*
	**%**	63.6%	0.0%	
**Yes**	**n**	4	12	
	**%**	36.4%	100.0%	

## Discussion

MN proved to be a sensitive (90.9%) and specific (100%) marker in OPMLs with dysplasia, whatever the grade was. The diagnostic accuracy of MN was significantly high with a confidence of 95.2%. That is in agreement with the case–control study by Grover et al. (2014) that demonstrated MN accuracy 88% in OPMLs [24]. Additionally, the cross sectional study conducted by Kumar et al. (2017) has documented nearly similar results; sensitivity 94.7% and specificity 91.4% [25].

The absence of MN in risk factors group as compared to OPMLs group suggests a link of this marker with early chromosomal instability as a result of a defect in DNA repair. Cancer risk factors usher in cumulative damage when it reaches its threshold; oral lesions start to appear corresponding to the molecular damage. MN evaluation is regarded as a first-level test for screening OPMLs to identify dysplastic or molecular alterations which would be an indication for histological control, even in clinically apparent benign oral lesions [26, 27].

On the other hand, the sensitivity of MN in malignant group was much lower (52.2%) than in OPMLs. Different results were previously obtained; the case–control study by Nanayakkara et al. (2016) recorded sensitivity 77% and specificity 98% for MN [28]. The cross-sectional study by Gupta et al. (2014) has revealed sensitivity 81.6% and specificity 68.4% for MN detection [29]. Moreover, the cross-sectional study by Sukegawa et al. (2020) has shown sensitivity 79.3% and specificity of 69.8% for MN [30].

Despite MN being a frequent observation in the early process of carcinogenesis, however, it might not be detected as the disease progresses when other nuclear changes become more pronounced as pleomorphism in nuclear shape and size. MN is not a passenger event in carcinogenesis, but it is an integral part in the events of DNA damage and tumor progression. MN are extra nuclear bodies incorporating damaged chromosome fragments or whole chromosome that were not included into the nucleus after cell division. The DNA in MN, being damaged, results in highly localized chromosomal rearrangements called “chromothripsis,” which in turn provoke oncogene amplification and tumor suppressor loss. MN contribute widely to many aspects of cancer biology, and the DNA in MN might just be the initiation of a cascade of genome instability [31]. Based on this background, the absence of MN in the cytological samples of malignant group does not rule out its detrimental effects on the disease progression but instead could indicate the escalation of cytological signs indicative of malignancy.

Cytological diagnosis of malignancy depends on the presence of a combination of cellular and nuclear alterations, but not necessarily the full spectrum of the morphologic abnormalities. There are no morphological features that can be used generally and consistently, in a mathematical algorithm, to reach a diagnosis of malignancy. Moreover, the same histogenetic type of cancer can have a wide range of morphological alterations, and also, the oncogenic abnormalities and the clinical presentations are found to be varied [6, 32]. In the present study, cellular pleomorphism was found in all OPMLs with dysplasia followed by prominent nucleoli and anisonucleosis (90.9%). Nuclear hyperchromasia was found in more than half of the study participants with OPMLs (63.6%). Similar data was reported by Bhandari and Gadkari (2015) who conducted a cross-sectional study on OPMLs and suspected malignant lesions [15]. The presence of cellular morphological diversity in all OPMLs with dysplasia indicated the high proliferation rate of dysplastic cells. Cell-to-cell variation is a sign of instability in the phenotype of the cells, and an unstable phenotype causes further genetic and epigenetic instability [14].

Increased cellularity and discohesiveness of cells were markedly obvious in almost all malignant lesions (95.7% and 91.3%, respectively). The reason behind that could be related to the genetic instability process, which is a principle event in cancer development that ushers in cells with genomic variations (genomic heterogeneity). The genetically unstable cells expand to produce clones of cells with different mutations. Loss of adhesion is an obvious feature in these cells due to the alterations in the physiology of integrin, desmosomes, and adherens junctions [33, 34]. The nuclear changes were significantly scored in all included malignant lesions; nuclear hyperchromasia was at the top of the highly observed changes by a percentage of 95.7%, followed by anisonucleosis (69.6%). The irregular chromatin pattern is highly predictive of malignant transformation as it represents the morphological expression of the distorted DNA content of the nucleus [6], while anisonucleosis describes the variations in the size of cell nuclei and the marked rise in nuclear size is a consequence of increased DNA synthesis by malignant cells [14].

The diagnostic precision of conventional oral cytology has been improved through implementation of new quantitative techniques as DNA cytophotometry and cytomorphology. The technique of DNA-image cytometry depends on optical quantification of chemicals included into the DNA using computer-assisted image analysis, while cytomorphology reflects the proliferative activity of cell population. These methods are expensive and have many laboratory errors regarding preanalytical, analytical, and postanalytical phases [35].

### Limitation

The conventional analysis of MN may fade away over time and make way for other methods such as DNA cytophotometry and cytomorphology.

## Conclusion

The inclusion of MN along with cytological analysis of OPMLs enhances the diagnostic potential of oral cytology for detection of early dysplasia. However, the visual scoring of MN is greatly time-consuming, and the results rely on subjective interpretation of nuclei and MN. The regular cytological features are more established, quantifiable, and easier for interpretation. Oral exfoliative cytology is recommended for biopsy site determination and monitoring of OPMLs.

## Supplementary Information

**Additional file 1.** 

## Data Availability

The datasets used and analyzed during the current study are available from the corresponding author on reasonable request.

## Abbreviations

AUC: Area under the curve
HPV: Human papillomavirus
MN: Micronuclei
NPV: Negative predictive value
OPMLs: Oral potential malignant lesions
OLP: Oral lichen planus
OSCC: Oral squamous cell carcinoma
Pap stain: Papanicolaou stain
PPV: Positive predictive value
WHO: World Health Organization
