# Supplementary figures and images for: Micronuclei detection in oral cytologic smear: does it add diagnostic value?
Source: J Egypt Natl Canc Inst. 2023 Sep 25;35:31. doi: 10.1186/s43046-023-00188-x (PMC13313968; doi:10.1186/s43046-023-00188-x)

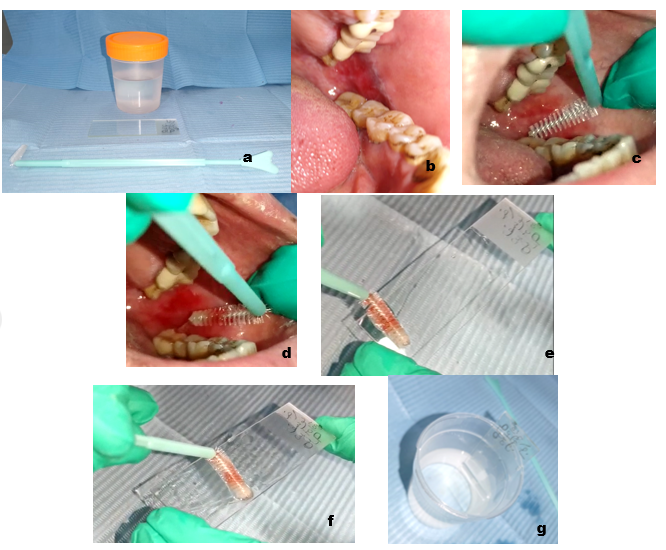


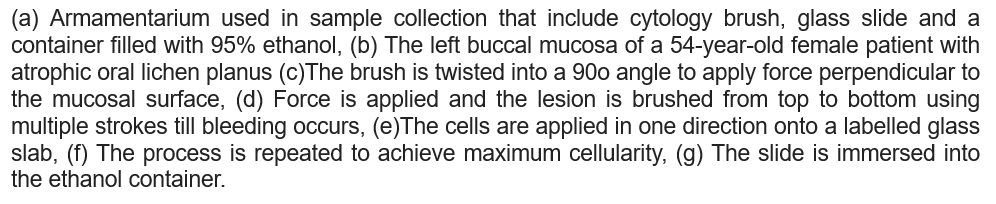

Supplement: Supplementary file 1 — Additional file 1. [file 43046_2023_188_MOESM1_ESM.docx]
